# Editorial on “Cell Therapy, Bispecific Antibodies and Other Immunotherapies against Cancer”

**DOI:** 10.3390/cancers15205053

**Published:** 2023-10-19

**Authors:** Vita Golubovskaya

**Affiliations:** Promab Biotechnologies, 2600 Hilltop Drive, Richmond, CA 94806, USA; vita.gol@promab.com

This Special Issue in *Cancers*, “Cell Therapy, Bispecific Antibodies and other Immunotherapies Against Cancer”, includes interesting reports and reviews on cell therapies and bispecific antibodies. The authors showed that cell therapy, bispecific antibodies, vaccine and immunomodulator approaches, combined with checkpoint inhibitors, are effective in improving anticancer therapies. The immunomodulators and immune checkpoint players, PD-1, PD-L1, CTLA-4, TIGIT and LAG-3, activate immune cells in the tumor microenvironment and increase immune response. The main approaches, challenges and future directions from this Special Issue are discussed.

Recently, a chimeric antigen receptor, CAR-T, cell therapy has revolutionized hematological cancer treatment. The FDA approved several CAR-T cell agents such as Kymriah, Yescarta, Tecartus and Breyanzi, which target CD19, and Abecma and Carvykti, which target BCMA antigens [1,2,3,4]. While the first round of treatments was successful, several challenges persist, such as low efficacy against solid tumors, cytokine release storm, the exhaustion of CAR-T cells, patient relapse, and the high cost of manufacturing. Novel generations of CAR-T cells are developed, such as bi-specific or tandem CAR-T cells [5,6,7,8,9,10], CAR-T cells with silenced checkpoint inhibitor pathways [11,12], CAR-T cells with different secreted cytokines (IL-15, IL-18, IL-12) to increase cell persistence and overcome a repressive tumor microenvironment, CAR-T cells with different switches to increase their safety. CAR-T cell therapy efficacy increased using different checkpoint inhibitors, such as PD-1, PD-L1, TIGIT, LAG-3, CTLA-4 and TIM-3 antibodies [11,13,14]. The disruption of PD-1 in CAR-T cells with Crispr/Cas-9 technology enhanced the functional activity of CAR-T cells [12]. Future combinations of CAR-T cells and other immunotherapies must be developed in clinical studies [15].

Another highly promising immunotherapy approach is CAR-NK cell therapy [16,17,18,19,20]. The advantage of using the NK cell for therapy is the absence of a GvHD (graft-versus-host disease) response applied to the generation of allogenic CAR-NK cells. CARs can be delivered into NK cells using lentiviruses, retroviruses, or mRNAs [21]. NK cells can be generated from different sources: blood PBMC, umbilical cord blood, induced pluripotent stem cells (iPSC) or the NK-92 cell line. There are several challenges for CAR-NK cell therapies: the low efficiency of expansion and genetic modification in vitro. CAR mRNAs can be embedded into lipid nanoparticles (LNPs) for increased stability and used for an efficient transfection of NK cells. Recently, an efficient encapsulation of BCMA and CD19-CAR mRNA into LNP and delivery to >500-fold expanded NK cells has been demonstrated, resulting in the generation of highly functional CAR^+^CD56^+^ NK cells against cancer cells and tumors [22]. The increased persistence of CAR-NK cells via a combination of CAR-NK cell therapy with other therapy approaches will be developed in the future.

Different immune cells, such as macrophages, T cell-infiltrating lymphocytes (TILS) [23], and gamma-delta T cells [24,25,26,27], can be used for the expression of CAR to target tumor cells. More clinical studies on these CAR-T cells are needed to understand their safety and efficacy against different types of cancer.

The application of T or NK cell-engaging bispecific antibodies is an alternative and promising approach to immunotherapy against cancer. BITE (blinatumomab), a CD19-CD3 antibody, is successfully used against B-cell malignant tumors in a clinical setting [28,29,30,31]. This year, the FDA granted accelerated approval for glofitamab, a CD20-CD3 bispecific antibody [32], against relapsed or diffuse large B-cell lymphomas (DLBCL). Many different designs of bispecific antibodies are developed with one domain binding to T/NK cells and another domain binding to cancer cell antigens: BITEs, Fc-containing, CrossMab, knob-hole, uni-, bi-valent and others [33,34]. There are several advantages of bi- and tri-specific T cell engagers versus cell therapy, such as off-the-shelf availability, easier logistics of administration and more economical manufacturing [35]. Several challenges exist for this approach, such as toxicity or repressive tumor microenvironment, that will be addressed in future pre-clinical and clinical studies. The improved engineering of antibodies and combination with inhibitors of tumor microenvironment can be applied to design these therapies.

Cancer vaccines (cell-based, peptide/protein-based, or gene-based) are another promising approach developed by several groups [36,37,38,39]. The goal of cancer vaccines is to target cancer cells via antigen-specific effector T cells. Activated T cells recognize MHC (major histocompatibility complex) I-peptide complexes, effector T cells target tumor cells and memory T cells prevent tumor relapse. The dendritic vaccine is a promising approach that stimulates T cells against tumor antigens [40]. The pulsing of DC with tumor cell lysate, tumor antigen or mRNA is a widely used approach for antigen delivery and the stimulation of an immune response. Dendritic cells are antigen-presenting cells and can be divided into several groups: conventional dendritic cells (cDC), plasmocytoid (pDC) and monocyte-derived DC (MoDC) [40]. The benefit of cancer vaccines is that they can target intracellular antigens versus CAR-T cells or bispecific antibodies, which target extracellular tumor-specific antigens [40]. Although conventional dendritic vaccines encounter limitations due to the low immunogenicity of cold tumors, the induction of immunogenic cell death (ICD) can convert cold tumors into hot tumors and improve DC vaccine potential. Immunogenic cell death can be achieved via chemotherapy, radiotherapy, photodynamic or photothermal therapy [40]. The local delivery of immunostimulants can increase the effect of immunogenic cell death of tumors and lead to the activation of DC and effector T cells.

The combination of cell therapy, bi-, tri-specific antibodies, CAR-NK, immunomodulators, checkpoint inhibitors and vaccines will be developed in future pre-clinical and clinical studies [41,42,43]. In addition, a personalized medicine approach will be used when patient tumors are sequenced to detect neoantigens that can be used for dendritic vaccine, bispecific antibody, and cell therapy development [35]. The combination therapy targeting several tumor antigens will be used to better target heterogeneous solid tumors. All discussed linked approaches are presented in Figure 1. For example, bispecific antibody (EpCAM-CD3 Ab is shown in Figure 1) can be delivered into tumors using mRNA [44], embedded into LNP [45] and attract T cells to kill tumor cells. The lysed tumor cells can release tumor neoantigens, serve as a cancer vaccine for attracting dendritic cells and, in combination with co-stimulants (cytokines, chemokines, receptor ligands and other immuno-stimulants), can target distant circulating tumor cells [37,46,47]. While EpCAM-CD3 mRNA-LNP was delivered intratumorally, future studies will expand the tumor-specific delivery of mRNA-LNP and the intravenous delivery of mRNA with tumor-specific expression of proteins. The proteins and antibodies can be produced inside tumors representing factories of proteins or antibodies, and secreted proteins will attract immune cells in the case of bispecific immune-engaging antibodies. Combination therapy with immunomodulators and checkpoint inhibitors will increase the efficacy of bispecific antibody and cell therapy to target distant metastatic tumor cells [48,49,50].

## Conclusions

Novel immunotherapy approaches, including bispecific antibodies, cell therapies, checkpoint inhibitors, vaccines and immunomodulators or their combination, must be developed and tested in future pre-clinical and clinical studies. The mRNA-LNP is a novel approach to deliver bispecific antibodies locally by enhancing immunomodulators to target distant tumor cells. The personalized medicine approach with a high-throughput sequencing of tumor antigens, detecting novel antigens and targets for immunotherapy, will be developed for more effective anticancer therapies. Dendritic, peptide and protein vaccines will be improved via novel tumor targets. The reports from this Special Issue in *Cancers* provide a basis to further develop novel immunotherapies.

## Figures and Tables

**Figure 1:**
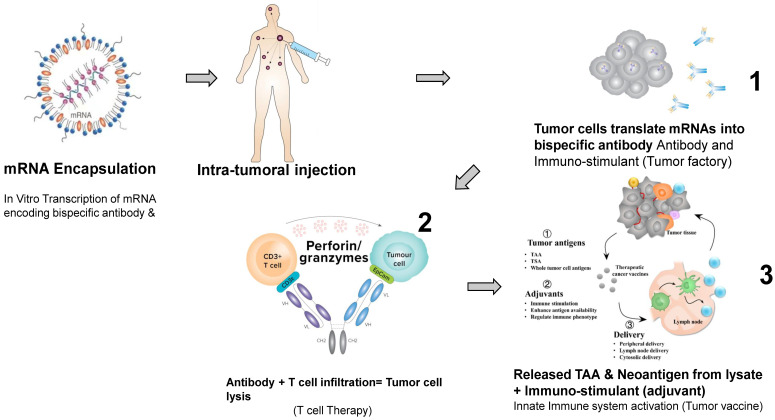
Bispecific antibody, immune T cell, immunostimulant and vaccine approaches. mRNA-LNP is shown for intratumoral delivery of bispecific antibodies. Secreted bispecific antibody attracts T cells to tumor and kills tumor cells. Tumor is lysed and serves as a tumor vaccine for stimulating further immune response in combination with immunostimulants.
